# Understanding Eustachian tube function^☆^

**DOI:** 10.1016/j.bjorl.2020.02.001

**Published:** 2020-02-28

**Authors:** Miriam S. Teixeira

**Affiliations:** University of Pittsburgh, School of Medicine, Department of Otolaryngology, Pittsburgh, United States

The primary function of the middle ear (ME) is to capture environmental sound pressures and transfer them to the cochlea. Efficient ME function requires that the ME be maintained at near-atmospheric pressure, which is achieved by periodic openings of the Eustachian tube (ET) and transfer of the gas bolus with the frequency sufficient to restore the near-atmospheric ME pressure.^1^ The ET is a biological communication between the ME and the nasopharynx, and is usually closed to prevent transmission of nasopharyngeal pressures, sounds and pathogens to the ME. During swallowing and other maneuvers, the activation of para-tubal muscles results in a transient opening of the ET lumen, allowing for free air circulation and equilibration of pressures between the nasopharynx and the ME. Although bacterial/viral infections, nasal allergies and other factors precipitate the development of ME inflammation, inadequate ET function causes the ME inflammation to persist long after the initial insult has been resolved. The traditional treatment for ET dysfunction (ETD) is insertion of a ventilation tube to bypass the ET and re-establish ambient ME pressure, resolve ME inflammation, clear effusions and thereby improve hearing.^2^

There is not yet a universally accepted definition for ETD, but with the emergence of new treatment modalities such as balloon dilation Eustachian tuboplasty (BDET), the need for clear diagnostic criteria for which this procedure would be indicated has become crucial. In the absence of such universally accepted criteria, many studies on BDET have relied on reported symptoms to diagnose ETD and recommend the ET dilation.^3^ Effort and continued research using objective measures are expanding the testing criteria, concepts and understanding of ET function.^4^, ^5^ Under this optic, ET opening mechanics has two main constituents: the passive constituents, represented by the elastic and tissue forces that keep the ET closed, and the active constituents, represented by the para-tubal muscles that oppose those forces to open the ET lumen.^4^, ^5^ Balance and harmony between these two components represents good ET function and dictates the extent and success in opening the ET to keep the ME healthy and at a near-ambient pressure. On the other hand, the selective or combined, partial or complete, simultaneous or temporal imbalance between these two components will create different degrees of abnormalities that range from patulous to obstructed ET.

The clinical application of this new approach to characterize ETD was presented in a recent publication in which 30 children (54 ears) from 6 to 17-years-old and with chronic and/or recurrent otitis media and findings suggestive of ETD were tested in a specialty clinic. Briefly, the ET passive function parameters were categorized based on the ET protective properties and included the opening pressure (OP), closing pressure (CP) and steady-state resistance (RS) for the Forced Response Test (FRT), and the degree of middle-ear pressure changes measured before and after sniff and Valsalva maneuvers. Active function parameters included dilatory efficiency (DE) for the FRT, percent correction of +200 daPa and −200 daPa ME–nasopharynx pressure gradients (with the Inflation–Deflation test for non-intact and with Pressure-Chamber tests for intact tympanic membranes) and ME pressure equilibrated before and after the Toynbee and Valsalva maneuvers. In addition, two derived parameters for the FRT were calculated: steady-state ET resistance (RS = steady-state pressure/trans-ET flow) and ET dilatory efficiency (DE = steady-state resistance/active resistance). Analysis of the ET function test data showed that both ET passive and active properties were normal in 3.7% of the ears, normal for passive/abnormal for active in 22.2% of ears, normal for active/abnormal for passive in 16.7% ears and abnormal for both parameters in 57.4% of the ears. In this population, it would be possible to identify ears in which BDET would be contra-indicated (those with normal active and passive properties and those showing easy to open ETs) and the ideal candidates for the procedure, those in which the necessary effort/pressures to open the ET were higher than expected.^5^

It is true that the sensitivity and specificity of the ET function tests have not yet been well established and that these initial studies need validation, but they are the first steps in recognizing that ETD is not just a state of being “too closed” or “too open”, but rather a spectrum of disorders that may have distinct pathophysiologies. The use of objective measures is extremely important not only to define the type of ETD but also determine the success of new treatment modalities. Among the challenges are the identification and development of new tests to better discriminate the passive and active properties of the ET and the establishment of the range of normality for each parameter. The goal is to create test protocols that are reproducible and simple enough to be used in a clinical setting, for example, by enhancing the capabilities of tympanometers' resident software that is already commercially available. The expectation is that a better understanding of the individual ETD characteristics will allow us to tailor the treatment according to the specific root cause and avoid unnecessary interventions.

## Funding

Supported in part by 10.13039/100000002National Institutes of Health Grant P-50 DC007667

## Conflicts of interest

The author declares no conflicts of interest.
